# Cognitive Processes Associated with Sequential Tool Use in New Caledonian Crows

**DOI:** 10.1371/journal.pone.0006471

**Published:** 2009-08-05

**Authors:** Joanna H. Wimpenny, Alex A. S. Weir, Lisa Clayton, Christian Rutz, Alex Kacelnik

**Affiliations:** Department of Zoology, University of Oxford, Oxford, United Kingdom; University of Cambridge, United Kingdom

## Abstract

**Background:**

Using tools to act on non-food objects—for example, to make other tools—is considered to be a hallmark of human intelligence, and may have been a crucial step in our evolution. One form of this behaviour, ‘sequential tool use’, has been observed in a number of non-human primates and even in one bird, the New Caledonian crow (*Corvus moneduloides*). While sequential tool use has often been interpreted as evidence for advanced cognitive abilities, such as planning and analogical reasoning, the behaviour itself can be underpinned by a range of different cognitive mechanisms, which have never been explicitly examined. Here, we present experiments that not only demonstrate new tool-using capabilities in New Caledonian crows, but allow examination of the extent to which crows understand the physical interactions involved.

**Methodology/Principal Findings:**

In two experiments, we tested seven captive New Caledonian crows in six tasks requiring the use of up to three different tools in a sequence to retrieve food. Our study incorporated several novel features: (i) we tested crows on a three-tool problem (subjects were required to use a tool to retrieve a second tool, then use the second tool to retrieve a third one, and finally use the third one to reach for food); (ii) we presented tasks of different complexity in random rather than progressive order; (iii) we included a number of control conditions to test whether tool retrieval was goal-directed; and (iv) we manipulated the subjects' pre-testing experience. Five subjects successfully used tools in a sequence (four from their first trial), and four subjects repeatedly solved the three-tool condition. Sequential tool use did not require, but was enhanced by, pre-training on each element in the sequence (‘chaining’), an explanation that could not be ruled out in earlier studies. By analyzing tool choice, tool swapping and improvement over time, we show that successful subjects did not use a random probing strategy. However, we find no firm evidence to support previous claims that sequential tool use demonstrates analogical reasoning or human-like planning.

**Conclusions/Significance:**

While the ability of subjects to use three tools in sequence reveals a competence beyond that observed in any other species, our study also emphasises the importance of parsimony in comparative cognitive science: seemingly intelligent behaviour can be achieved without the involvement of high-level mental faculties, and detailed analyses are necessary before accepting claims for complex cognitive abilities.

## Introduction

In comparison with other animals, there can be no doubt that humans are both exceptionally intelligent and outstanding in the intensity and complexity of their tool use. It is likely that both traits have evolved in unison, although we can only speculate about the direction of causality. In humans, one possibility is that tool use promoted the evolution of exceptional intelligence, without requiring high intelligence to get started. From a comparative perspective, this would imply that differences between species in traits associated with tool use may be due to differences in the specific ecological conditions that make tool use advantageous, possibly leading to the evolution of motivational rather than cognitive differences. The presence of advanced cognitive functions in species distinguished for their sophisticated tool-oriented behaviour should still be considered as a working hypothesis [1].

**Figure 1:**
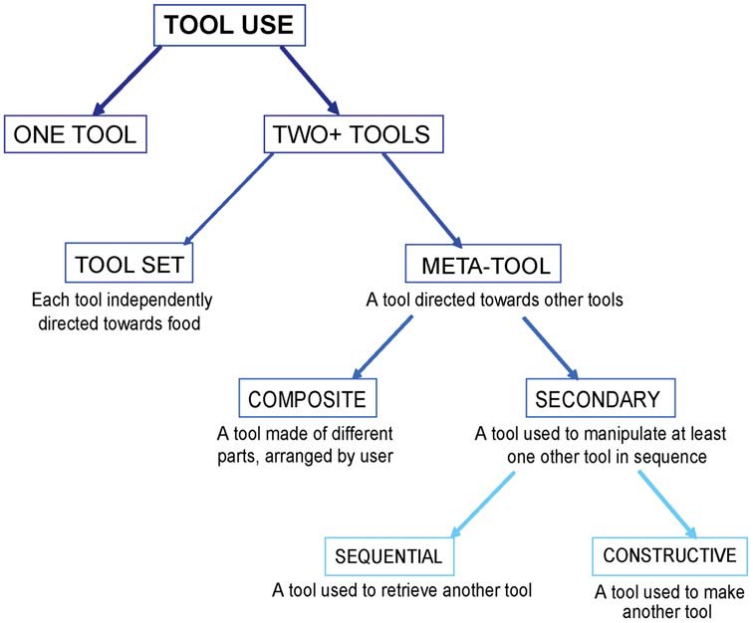
Proposed terminology for classifying different types of animal tool use. The present study investigated sequential tool use, which falls under the broad category of ‘meta-tool use’, and more specifically can be thought of as a type of ‘secondary tool use’. Depending on the number of tools used, sequential tool use can be further divided into two-tool, three-tool, and *n*-tool sequences. For a detailed discussion of terms, see main text.

Many non-human animals are known to use and make tools [2]. However, there is considerable variation in the frequency and complexity of tool use, even between closely related taxa, and attempts to explain this variation face the challenge of distinguishing between several plausible hypotheses, of which higher cognitive proficiency is only one candidate. It may be that exceptional tool users owe their skills to unusual cognitive abilities; on the other hand, it may be that even the most impressive demonstrations of tool use may be achieved by cognitive processes common to many animals that do not regularly use tools. To address the potential complexity of the cognitive processes involved in tool use, it is useful to examine instances of tool-oriented behaviour that, at least at first sight, appear to be highly sophisticated [3]. One example of this is the ability to modify material appropriately in order to use it as a functioning tool [4], [5]. Another is ‘secondary tool use’–using one tool on another (non-food) object to access it or modify it for use as a tool. The latter is the focus of this paper, and since it has been referred to with a number of different names, we start by providing a systematic classification of current terminology (see Figure 1).

Very similar behaviour has been described in different studies as ‘meta-tool use’, ‘secondary tool use’, ‘sequential tool use’ and as use of a ‘tool set’ [6]–[10], or examined without a specific name [11]–[14]. We begin by isolating the latter term, ‘tool-set’, from the others, and from the behaviour which is the focus of this paper. We believe that ‘tool-set’ is best reserved for occasions where more than one object is used in a sequence, but with the distinction that each of these actions is aimed towards the food (or food-containing object). For example, chimpanzees (*Pan troglodytes*) use a stout stick to puncture a termite mound and then a more slender and flexible one to extract the prey [10], [15]–[17]. In contrast to this, the other terms refer to a class of behaviours in which tools are directed at objects that are not the agent's ultimate goal (normally food), but which have a role in achieving the ultimate goal–for example, when a tool is used to retrieve another tool. We propose that ‘meta-tool use’ should be used as an umbrella term to cover all instances of this type of tool use. Within meta-tool use, there are two subcategories: ‘secondary tool use’ and ‘composite tool use’. A composite tool can be defined as a tool composed of a number of distinct parts, with those parts arranged by the user [9]. To our knowledge, the only potential observations of composite tool use in the wild are those by Matsuzawa [9], [18], who saw chimpanzees use a stone as a ‘wedge’ to make the surface of another stone level, so that it could subsequently be used as an ‘anvil’ for cracking nuts (but see [19] for an argument as to why the wedge may not fulfil the criteria to be called a ‘tool’). In contrast, secondary tool use refers to occasions where one tool is used to act upon another object (itself destined to become a tool) that is eventually used on the ‘ultimate goal’. This would include tools used to make other tools, or to retrieve objects to be used as tools. Using tools to make tools is how the term secondary tool use is commonly used in the literature on hominid technology [6]; however, we propose that this term should be subdivided to also include the use of tools to retrieve other tools. We refer to the former as ‘constructive’ and the latter as ‘sequential’ tool use (see Figure 1). The complexity of sequential tool use might reasonably be expected to be more demanding as the number of stages between initiation of the sequence and acquisition of the final goal increases. There are, of course, other classification possibilities, but we hope that ours has the virtue of being descriptively explicit and of separating classes of acts of potentially different levels of cognitive demand. Throughout this paper, we use this classification, and focus in particular on sequential tool use, i.e. the use of tools to retrieve other, out-of-reach objects that will serve as tools. In our experiments, subjects received different conditions, which we labelled to reflect the nature of the task. Notice that while we label the tasks, the tools themselves are simply described by their location at the start of the trial rather than as ‘primary’ or ‘secondary’, because the same object may, at different moments, be used to achieve different goals. That is, a short tool may be used to retrieve food if it is within reach, but on a different condition may be used to retrieve other tools. Labelling the tools according to their function is therefore impossible until after the action has been performed.

The rarity of secondary tool use in non-humans, and its relatively recent appearance in the human fossil record, has led some authors to assume a strong association with the ability to plan ahead and act on the basis of reasoning (for example [6], [8]). To explore whether non-human animals are capable of secondary tool use in captivity, several researchers have focused on the following problem. A subject is presented with a food reward that is out of reach. At the same time, it is presented with a readily available tool that is too short to reach the food, but sufficiently long to obtain another out-of-reach tool; only the second tool can reach the food. The solution is a clear example of sequential tool use: the subject should use the available tool first, to reach for the out-of-reach but suitable tool, and then use the latter to reach for the food. At least some individual chimpanzees [14], [20], [21], gorillas (*Gorilla gorilla*) [13], orangutans (*Pongo pygmaeus*) [13], and capuchin monkeys (*Cebus* spp.) [11], [12], [22] are able to perform this behaviour spontaneously, and macaques (*Macaca* spp.) [7], [12] and cotton-top tamarins (*Saguinus oedipus*) [23] can acquire it after considerable training. More recently, members of two corvid species have also been shown to use two tools in a sequence [8], [24]. Bird and Emery reported that non tool-using rooks (*Corvus frugilegus*) spontaneously used tools under a variety of circumstances, including what the authors termed ‘metatool use’, where subjects dropped a large stone into a container to release a small stone, which was then used to acquire food [24]. Taylor *et al.* [8] reported that New Caledonian crows (*Corvus moneduloides*), known to be flexible tool-makers and users in the wild [25], were able to extract a long tool from a tool-box using a shorter tool, and then use the long tool to retrieve food. The authors argued that this behaviour demonstrated ‘analogical reasoning’, by which they meant that the subjects inferred, by analogy with food-retrieval, that a tool could also be used to retrieve another tool, rather than just food.

Demonstrating sequential tool use in non-human animals is impressive, and might indeed expose the ability to plan or reason about problems, but none of the experiments to date have explicitly examined whether such advanced cognitive processes are actually involved. There are several possible explanations for sequential tool use that do not invoke goal-directed behaviour, planning, causal understanding, or analogical reasoning (for a discussion of the conceptual issues surrounding this terminology, see [3]). For example, subjects might attempt to retrieve the food with the available tool, but after failing to do so might perform various ‘displacement activities’ such as probing randomly elsewhere, and thereby extracting the usable tool as they would any inappropriate object in the vicinity. Another possibility is that, if subjects had previously learned separate components of the sequence (for example, using a tool to probe for food, and extracting tools directly from the container in which tools would later be out of reach), they could ‘chain’ these into a single sequence. Such chaining of previously learned behaviours was observed in pigeons by Epstein *et al.* [26], in a famous replica of Köhler's [14] study with chimpanzees, where some subjects stacked boxes to reach an otherwise unobtainable reward. Epstein *et al.* used pigeons that were trained, in separate sessions, to climb on a box to peck a banana-looking key (the box was fixed under the key), and to push a box to a green spot on the floor (no banana key was present, and climbing on the box was not reinforced); when the box was placed away from the banana key and no green spot was present, the subjects spontaneously pushed the box until it was underneath the banana key, climbed onto it and pecked the banana key. In contrast, subjects that had not been trained on all components of the task never solved it (for example, those birds trained only to push the box and not to climb on it, or those trained only to climb on the box and not to push it).

Because of their design, previous experiments on sequential tool use could not distinguish between goal-directed, planned behaviour and simpler, more parsimonious explanations. Firstly, in most of the studies [7], [13], [20] there was only one unreachable object (the tool required to retrieve food), and few or no control conditions; consequently, retrieval of the out-of-reach tool could well have resulted from misdirected (or playful) probing with the available tool as a consequence of the subject's inability to reach the food with the latter. Taylor *et al.* [8] presented a stone as well as an out-of-reach tool, and since their subjects rarely extracted the stone this demonstrates that tool extraction was not entirely random. However, choosing to retrieve a stick over a stone may be the result of differential stimulus salience rather than anticipated use of the retrieved object. Compared to sticks, stones are not typically used by crows to obtain food and hence they are probably not the target of their attention. Bird and Emery also presented their rooks with a choice of two out-of-reach tools; in this case both options were stones that had been previously rewarded, so the correct choice shown by their subjects was more revealing [24]. However, in their experiment, it was still the case that one of the options was always correct, which may have been a simple discrimination to learn. Secondly, in most studies [7], [8], [12], [13], [27], where sufficient detail is provided to be able to tell, subjects were pre-trained in the main components of the task before testing. Subjects were usually trained to pick up and use tools from the location where they were later placed out of reach during the test, and most were also trained to use the available tool to retrieve food. Thus, like Epstein *et al.*'s pigeons [26], they were trained in each component of the task separately, and might have been ‘chaining’ these together just as the pigeons did; the extracted tools could have become secondary reinforcements worth extracting *per se* rather than with the goal of using them as tools [28]. Thirdly, the inaccessible tool was often placed either adjacent to the reward or between the subject and the reward [7], [12], [20], [23], [27] increasing the probability of subjects retrieving it by chance, while attempting to retrieve the food with the unsuitable but available tool. Finally, tasks were often introduced in a stepwise manner, progressing from the easiest to the most difficult conditions, which might have had the effect of training the subjects by reinforcing simple sequences of behaviour generated through random processes [8], [12], [13], [27].

In this study, we examined the cognitive processes that underlie sequential tool use in New Caledonian crows, introducing experimental conditions that allow us to discriminate between the aforementioned possibilities. Our experiments included the following unique features:

1. Subjects were presented with multiple inaccessible tools, which differed in functionality and were spatially and visually separated from the food reward. If tools are extracted because they have become secondary reinforcers due to previous experiences, then we might expect them to be extracted independently of their functionality, but if the crows plan their sequence of actions they should be selective according to present needs [29].
2. There were several, intermixed conditions where the position of food and/or tools determined what sequence of behaviour was required for success: in one condition we required subjects to retrieve and use three tools in sequence, something never demonstrated in a non-human animal without specific training. Use of three tools in correct order is much more challenging than the use of two tools for two reasons. Firstly, the initiation of the sequence is more remote from the goal, thus requiring greater abstraction. Secondly, since the generation of three actions in a correct sequence at random is less probable than that of two, it is much more difficult to acquire the behaviour by reinforcement of randomly generated sequences.
3. We included control conditions where: (i) food was not present; (ii) inaccessible tools were replaced by other unusable objects; or (iii) sequential tool use was not required. If crows still extracted tools and probed into the food container when food was not present, it would be difficult to conclude that successful sequences of behaviour when food *was* present were the result of goal-directed planning. Similarly, extraction of non-functional objects from the tool-dispensing apparatus (when food was present) would argue against rational planning being the explanation for tool-extraction during experimental conditions.
4. Finally, we manipulated the degree of pre-training the subjects received. If successful sequential tool use is dependent on behavioural chaining (see above) then only subjects who received experience with all components of the task should succeed.

## Results and Discussion

### Experiment 1

**Figure 2:**
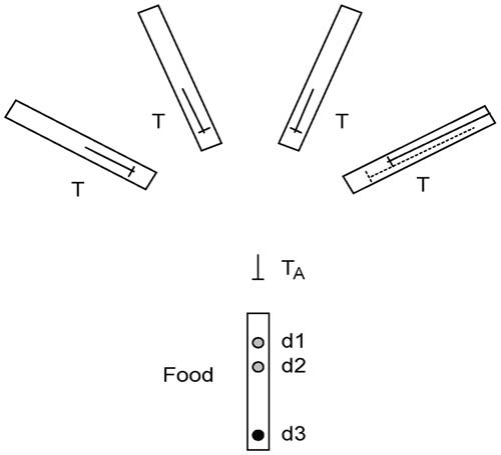
Schematic of the apparatus used in Experiment 1 (seen from above). The food reward is located in the central food-tube, and can be at any of three depths (d1, d2 or d3). Tools are located out of reach inside each of four additional tool-tubes (denoted T). Three of these tools are 10 cm in length and one is 20 cm. None of them are accessible by beak alone. The only tool that is directly manipulable is the tabletop tool (T_A_), which is 6 cm long. The trial-type depicted is ‘Tertiary’; i.e. the food is at its deepest, and the longest tool is in the rightmost tool-tube, out of reach of either beak or tabletop tool (the position of the longest tool on all other conditions is shown by the dashed line in the same tube). The correct sequence of behaviour is for the subject to probe for any 10 cm tool with the tabletop tool, then use the 10 cm tool to probe for the 20 cm tool (in the right hand tube), and finally use the 20 cm tool to probe for food.

In this experiment, we investigated whether six New Caledonian crows would spontaneously use an available tool to retrieve tools placed out of beak reach, when these were necessary to retrieve a food reward. The apparatus consisted of one transparent ‘food-tube’ and four transparent ‘tool-tubes’, which were positioned such that subjects were facing away from the food-tube when interacting with tool-tubes (see Figure 2). During the experiment, one long and three medium-length tools were positioned out of beak reach within the tool-tubes, while a short tool was freely available on the table (termed the ‘tabletop’ tool). There were three experimental and two control conditions (Table 1), with each subject initially receiving three or four trials per condition in pseudo-random order (trial numbers were deliberately low, to minimize the amount of learning during the experiment). The sequence of behaviour necessary to retrieve the food was dictated by the depth of the food and tools within the tubes, with the most demanding condition requiring the use of three tools in a sequence (‘Tertiary’; see Table 1 for an overview of all conditions). Subjects received different pre-testing experience, to investigate whether crows required experience of extracting tools from tubes with their beaks to perform successfully in the subsequent sequential tool use tasks. Thus, prior to experimental trials, three subjects were allowed to retrieve tools from tubes with their beaks and use these tools to retrieve food (‘experienced’ subjects: ‘Betty’, ‘Pierre’, and ‘Uék’), whereas three were exposed to empty tool-tubes and a freely-available tool that could be used for food retrieval (‘inexperienced’ subjects: ‘Barry’, ‘Kenny’, and ‘Nalik’). To avoid handling stress [30], the birds lived in pairs in free flying aviaries and entered the test room voluntarily. Due to the presence of substantial qualitative individual differences in behaviour we describe individual results rather than averaging quantitative indices of performance across individuals.

**Table 1:** Description of conditions for Experiments 1 and 2.

Condition	Condition type	Experiment	Food depth	Trial description	Most efficient behaviour
*Primary*	Control	1, 2	7 cm	Food within reach of the (longer) tabletop tool.	Probe food with the (longer) tabletop tool.
*No-Food*	Control	1 (except Betty and Pierre), 2	NA	No food present.	Do not probe for anything.
*No-Tools*	Control	2	15 cm	Food at 15 cm, tools replaced by non-tool objects.	Do not probe for anything.
*Secondary-Any*	Experimental	1, 2	13 cm	Food out of reach of the tabletop tool, but within reach of any out-of-reach tool.	Probe for any tool with the (longer) tabletop tool. Use extracted tool to probe for food.
*Secondary-Long*	Experimental	1, 2	25 cm	Food only reachable by the longest out-of-reach tool.	Probe for longest tool with the (longer) tabletop tool. Use longest tool to probe for food.
*Tertiary*	Experimental	1, 2	25 cm	Experiment 1: Food only reachable by the 20 cm out-of-reach tool, which is reachable with any other out-of-reach tool, but not the tabletop tool. Experiment 2: Food only reachable by the 25 cm tool, which is reachable only by the 20 cm out-of-reach tool.	Experiment 1: Probe for any out-of-reach tool with the tabletop tool. Use the extracted tool to probe for the 20 cm tool. Use 20 cm tool to probe for food. Experiment 2: Probe for the 20 cm tool with the longer tabletop tool. Use 20 cm tool to probe for 25 cm tool. Use 25 cm tool to probe for food.
*Length-Only*	Control	2	7 cm 13 cm 25 cm	These trials correspond to the Primary (7 cm), Secondary-Any (13 cm) and Secondary-Long/Tertiary (25 cm) trials, as described above, with the exception that tools in the frame are within beak range and do not need to be probed for.	7 cm: Probe for food with the longer tabletop tool. 13 cm: Probe for food with any tool from the tool-frame. 25 cm: Select the longest tool from the tool-frame. Use tool to probe for food.

Sequential tool use is required in experimental, but not control conditions. Note that in Experiment 2 there were two tabletop tools of different lengths, compared with just one in Experiment 1.

The main finding was that all three experienced subjects showed sequential tool use on their very first experimental trials (Secondary-Any, Secondary-Long and Tertiary), and consistently thereafter. In contrast, the inexperienced subjects only used a tool to extract another on four (pooled) of their first 20 trials, and never retrieved food in the experimental conditions (i.e., when more than one tool was required). Consequently, we discuss the performance of the two groups separately.

#### Experienced subjects

As shown in Table 2, Betty retrieved food on all her trials, and was the only subject to succeed on the Tertiary condition (Video S1). The other two subjects successfully retrieved food under both two-tool conditions, but, although they often extracted more than one tool on Tertiary trials, they never used them in the correct sequence to acquire food. Interestingly, on two Tertiary trials Pierre initially tried to extract an appropriate tool with the available tabletop tool, but after failing to do so, he left the room and returned moments later with a natural twig tool (longer than the tabletop tool). He then used these twig tools to extract the longest tool (after first briefly probing for food on one trial), with which he retrieved the food reward. Thus, by finding a suitable object outside the confines of the experimental set up he transformed the task from requiring three tools to two, and then proceeded to solve it appropriately. He also left the chamber and brought in his own tool on one Secondary-Long trial (Video S2), and on one Secondary-Any trial he brought a tool in at the start of the trial and probed directly for the food.

**Table 2:** Overview of crows' success across conditions in Experiment 1.

Group	Subject	Primary	Secondary-Any	Secondary-Long	Tertiary	Number of trials per condition
Experienced	*Betty*	3	3	3	3	3
Experienced	*Pierre*	3	2(1)	1(1)	0(2)	3
Experienced	*Uék*	4	4	2	0	4
Inexperienced	*Nalik*	2	0	0	0	4
Inexperienced	*Barry*	3	0	0	0	4
Inexperienced	*Kenny*	4	0	0	0	4
Inexperienced	*Barry2*	9	5	2	0	9
Inexperienced	*Kenny2*	14	0	0	0	14

Pierre sometimes brought his own tools into the testing chamber to extract tools when he failed to extract them using the tools provided; the number of trials on which this happened is shown in brackets. Barry and Kenny both received additional testing trials (Nalik had died at this point) as described in the main text; these are presented as ‘Barry 2’ and ‘Kenny 2’.

We examined three aspects of the subjects' behaviour which relate to the cognitive processes underlying their performance: (i) whether subjects attended to the distance to food when choosing where to probe with the tabletop tool; (ii) whether they were selective about the tools that they used to probe for food; and (iii) whether they still extracted tools when there was no food to be extracted.

We investigated whether subjects attended to the position of the food by examining whether their first probe with the tabletop tool was aimed at tools or at food. All three subjects immediately used the available tool to extract food in all except one of their Primary trials. In the experimental conditions, when a completely rational strategy predicts not even trying for the food with the tabletop tool, probing instead directly for one of the inaccessible but potentially suitable tools, Betty appropriately probed into tool-tubes first in 8 of 9 trials; in contrast, Pierre and Uék first briefly probed into the food-tube first on 8/8 and 7/12 trials, respectively, but after these unsuccessful probes both subjects quickly probed into the tool-tubes (median interval between probing for food and probing for tools: Uék, 1.9 s; Pierre, 1.1 s). These brief inappropriate probes could reflect an inability to inhibit probes towards the food, or they could have been depth gauging actions.

To explore tool selectivity, we looked at the first extracted tool that the subjects used to probe for food. While the (deliberately) low number of trials per condition means that tool choice cannot be analysed in detail, subjects did not appear to choose in advance the tools they required. On the Secondary-Long condition (where only one of the four out-of-reach tools was suitable), the subjects showed weak evidence for selectivity, using the correct, longest tool to probe first for the food on 5 of 10 trials (pooled across subjects; binomial test; *z* = 1.83, *p* = 0.08). On the Tertiary condition, they used the correct, longest tool to first probe for the food on only 2 out of 10 trials (*z* = −0.37, *p* = 0.76).

Finally, we investigated Uék's behaviour on the No-Food control condition, which was introduced after Pierre and Betty had completed testing and were unavailable for replicates. We reasoned that, if tool extraction on experimental trials was goal-directed and related to retrieving food, in the No-Food control condition, subjects should neither extract further tools nor probe the food-tube. Uék never probed the empty food-tube with the tabletop tool, but she did use it to extract further tools on all trials and used these to probe into it. This might suggest that, when food was present, her extraction of tools is inconclusive as evidence that she was directly driven by her goal to extract the food. However, we make this comment with caution, for even if the tools had acquired some value over the course of experimentation, she may still have been goal-directed in her extraction of tools during testing; the two possibilities are not mutually exclusive. Even though this control was only run in a single experienced subject, the result suggests that such goal-absent or goal-modified controls might provide important information in establishing the goal-directedness of sequential tool actions.

#### Inexperienced subjects

All three inexperienced crows obtained food with the tabletop tool where this was possible, but failed in all tasks where tools had to be extracted (Table 2). Furthermore, some other aspects of their behaviour argue against the interpretation that these subjects were reasoning about the task: one subject (Nalik) repeatedly interacted with tool-tubes, and on three trials used the available tool to obtain further tools, but he never then made use of these for food extraction. On the No-Food trials, the empty food-tube was probed in two trials (out of a pooled twelve). Barry probed with the tabletop tool, but Nalik used that tool to extract another and probed the empty tube with it; this was the only occasion when an inexperienced bird used the available tabletop tool to extract a tool that was then used to probe into the food tube.

To explore whether the inexperienced birds' failure to show successful sequential tool use was a result of limited experience, we gave two of them (Nalik had died) additional blocks of trials. One subject (Barry) extracted a tool on his second additional trial (22nd trial overall; 14th experimental trial overall), and subsequently repeatedly interacted with tool-tubes, showing performance levels similar to those observed in two of the experienced subjects, Uék and Pierre (7/18 successes in Secondary-Any and Secondary-Long trials; like Uék and Pierre, he never solved the Tertiary task). In contrast, even after a total of 77 extra trials, the other bird (Kenny) never used one tool to retrieve another. Further tests suggested that his poor performance was due to lack of attention or aversion to the tool-tubes: even when tools protruded *out* of the tubes, and could be obtained by beak, Kenny did not retrieve them, although he continued to retrieve the food when it was within reach of the tabletop tool.

#### Discussion

In summary, in this first experiment one of our subjects (Betty) provided the first observation of spontaneous three-tool sequential tool use in a non-human animal, and overall four of the six birds were successful in using an available tool to retrieve inaccessible tools that were then used to extract food. The difference between naïve and pre-trained animals suggests that elements of the pre-testing procedure might contribute to successful behaviour, an observation that runs against Taylor *et al*'s [8] interpretation that analogical reasoning (i.e., generalising from using tools for food retrieval to using them to retrieve other objects) can be inferred from sequential tool use. If crows know that they can use tools to obtain food, they do not necessarily make use of this knowledge to deduce that tools can be used to obtain tools. Even in the experienced subjects, we found no reliable evidence of detailed planning of the sequence of tools–they seemed to know *when* to extract further tools, but not *which* ones. Interestingly, specific pre-training experience was neither always required, nor always sufficient, since one inexperienced subject (Nalik) did extract inaccessible tools in the initial set of trials (although not appropriately to obtain food), another (Barry) showed sequential tool use in his second testing session, and yet another subject (Kenny) never did so even after rather extensive experience.

While the ability to plan or reason by analogy has not been demonstrated, two main reasons make the opposite conclusion premature (namely, that New Caledonian crows are incapable of either of these). Firstly, given that New Caledonian crows have previously been shown to choose appropriately between directly available tools [29], it is possible that the design of the apparatus from which the tools had to be extracted impaired subjects' ability to judge their lengths and select accordingly. Surface reflections from the acrylic tubes containing the tools may have obscured the end of the tools when viewed from the side, and perspective may have made distinguishing between tools of different lengths difficult when viewing from the front. Since subjects could not perch on top of the tubes to view the tools from above they would have had to look into each tube and remember the length of each tool within, which might have hindered their selection. This seems plausible, since in a previous study where the crows did show length selectivity [29] potential tools were offered vertically, next to each other, and individuals could manipulate them before approaching the food-containing apparatus. Secondly, while the failure of the inexperienced subjects may have been due to their lack of experience at extracting tools from tool-tubes, and therefore their inability to ‘chain’ *sensu* Epstein *et al.* [26], it is also possible that the reason for the difference between the groups was that experienced subjects had learned to pay *attention* to tool-tubes. Inexperienced subjects had never been reinforced for interacting with the tubes during training, and so might have learned their irrelevance and ignored them during testing. We carried out a second experiment to address these issues.

### Experiment 2

**Figure 3:**
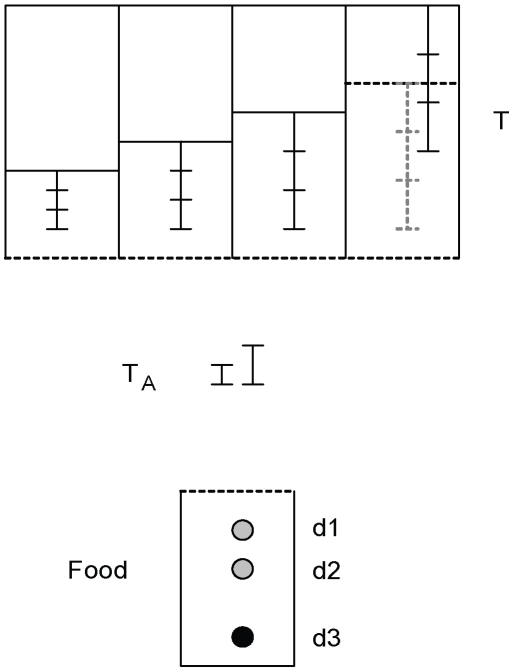
Schematic of the apparatus used in Experiment 2 (seen from above). The food reward is located in the smaller food-frame, and can be at any of three depths (d1, d2 or d3). Out-of-reach tools are located inside each of four lanes of a larger, mesh-bound tool-frame (denoted T). Tool lengths are 10, 15, 20 and 25 cm. None of them are accessible by beak alone. The only tools that are directly manipulable are the two tabletop tools (T_A_), one of which is 5 cm and the other 7 cm long. The trial-type depicted is ‘Tertiary’; i.e. the food is at its deepest, and the longest tool is out of reach of both tabletop tools (the dashed line shows the normal position). The correct sequence of behaviour is for the subject to pick up the longer of the two tabletop tools and probe with it for the 20 cm tool (located in the third lane from the left), then use the 20 cm tool to probe for the 25 cm tool (located in the far right lane), and finally use the 25 cm tool to probe for food.

This experiment was conceptually the same as Experiment 1, but we modified the procedure and apparatus to allow more detailed examination of the cognitive processes involved. We re-tested two subjects from the experienced group in Experiment 1 (Pierre and Uék; Betty had died) and another, naïve individual (Corbeau) with a new apparatus, additional control conditions, a new familiarization regime, and more trials per condition. To reduce the possibility that the acrylic tubes interfered with crows' perception of food depth or tool lengths, we replaced them with wire-mesh frames on which subjects could stand to view both food and tools directly (instead of through acrylic) from above (Figure 3). We also included two control conditions—‘No-Food’ (the food-frame was empty; this condition had been presented to four subjects in Experiment 1) and ‘No-Tools’ (unsuitable non-tool objects were placed in tool-frames instead of tools; see Table 1)—to reveal whether subjects would extract tools or non-tool objects even when this would not lead to the acquisition of food. All three birds received the following familiarization procedure: the food- and tool-frames were presented with food and non-tool objects (stones, within beak range) in all lanes, but never contained tools. This ensured that the subjects were reinforced for interacting with the tool-frame, but not trained to extract tools from it or to probe into it with tools (for details, see Materials and Methods). The testing protocol was similar to Experiment 1, except that subjects received at least 9 trials of each of the 6 conditions (in blocks of 6 trials, one per condition in randomized order). After these 54 trials, they all received at least 11 additional trials of the three-tool (Tertiary) condition. The inaccessible tools were of four different lengths (rather than just medium or long, as in Experiment 1), and we provided two tabletop tools rather than one, only one of which was sufficiently long to retrieve either food or further tools (see Figure 3). Following these sequential trials we carried out a similar experiment with the same individuals, but where sequential tool use was unnecessary (termed ‘Length-Only’ trials). The apparatus and experimental protocol were the same as that used in sequential trials, except that the tools were not pushed out of reach and were therefore accessible by beak alone. The purpose of this control was to determine whether tool selection was consistent in both types of trial; if crows choose inappropriate tools more often in sequential than non-sequential trials, this may indicate that sequential tool use imposes higher cognitive demands.

#### Sequential trials

**Figure 4:**
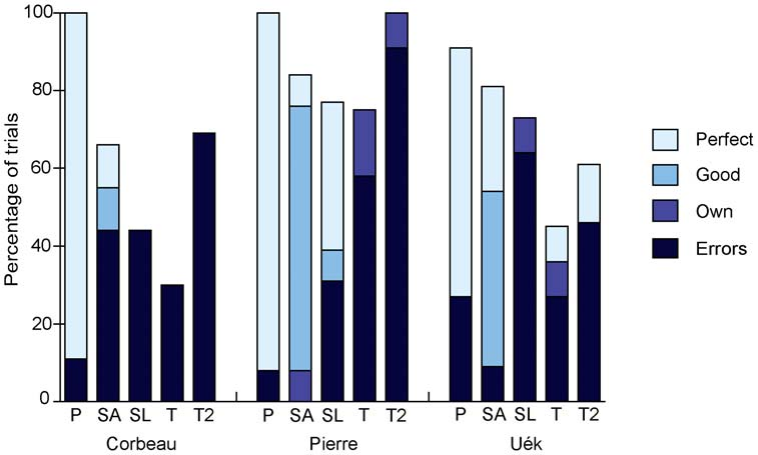
Success rates of crows in Experiment 2. Bar shading indicates the type of success: when ‘Errors’ were made, food was retrieved but the sequence of behaviour contained errors; ‘Own’ refers to a small number of trials where subjects used their own method of obtaining food (see text and Video S2); ‘Good’ refers to trials where the food was out of reach of the tabletop tool and subjects still directed their first probes into the food-frame, but all subsequent actions were correct; ‘Perfect’ means no errors were made in the acquisition of food. Trial types are coded as follows: P = Primary; SA = Secondary-Any; SL = Secondary-Long; T = Tertiary; T2 = Tertiary-2.

All three subjects probed for tools on their very first trial and consistently thereafter, with Pierre and Corbeau extracting a tool from the frame on their first experimental trial (Tertiary), and Uék on her fifth (Secondary-Long; Uék had difficulty actually extracting the inaccessible tools on earlier trials). Pierre first obtained food using an extracted tool on his seventh experimental trial (Secondary-Any), Corbeau on his fourth (Secondary-Any) and Uék on her fifth (Secondary-Long). All obtained food on every Primary trial, but their success rates in other conditions decreased as task complexity increased (Figure 4). All three subjects solved the three-tool problem (Tertiary; see Video S3) on at least some trials, and all had higher rates of success on the second block of these trials (Tertiary-2; Corbeau: 30% *vs* 69%, *n* = 23 trials; Pierre: 70% *vs* 100%, *n* = 20; Uék: 40% *vs* 62%, *n* = 23). There were relatively few ‘perfect’ performances on experimental trials (i.e., where subjects made no errors), with Uék being the only subject to show perfect performance in a Tertiary trial (Figure 4). As in Experiment 1, there were some instances where subjects retrieved the food using their ‘own’ technique, such as bringing their own tools into the testing chamber, or probing down through the mesh on the top of the food-frame; for descriptive purposes these six trials are included in Figure 4 but because of their lack of qualitative conformity to the main data set they were not included in subsequent quantitative analyses. Crows preferentially chose the correct, longer tabletop tool on almost every trial (Corbeau: 90%; Pierre: 92%; Uek: 88%; binomial tests: p<0.001 for all), with little variation across conditions.

As previously, we investigated whether crows probed first into the food- or the tool-frames, and with which tool. We also examined: (i) whether subjects exchanged tools at random; (ii) whether they extracted tools/objects from the frame or probed for the food in control conditions; and (iii) whether their performance on tertiary tasks improved with experience.

All three subjects nearly always probed for food before probing for tools in Primary trials, when the food was within reach of the tabletop tool (Corbeau: 89%; Pierre: 92%; Uék: 73%). In contrast, in conditions where the distance to food was greater, they first probed for tools more frequently, with no subjects first probing for food in the final block of Tertiary trials. We confirmed this statistically with a binary logistic regression, with ‘food depth’ and ‘subject’ as factors and ‘location of first probe’ as the dependent variable (towards the food, or the tools). As the distance to the food increased, the likelihood of subjects first probing towards the tools increased (*z* = 5.29, *p*<0.001). Furthermore, the same analysis revealed a significant difference between subjects, in the location of their first probes (*χ* ^2^ = 14.21, *df* = 2, *p* = 0.001): Uek was significantly more likely than Corbeau to first probe towards the tools (*z* = 3.43, *p* = 0.001), whereas there was no difference between Pierre and Corbeau (*z* = 0.42, *p* = 0.67). Corbeau and Pierre probed for food first on the majority of their Secondary-Any trials (77.8% and 91.7%, respectively), when food was at an intermediate distance, suggesting that they had difficulty estimating precisely how far they could reach with the tabletop tool; in contrast, they (and Uék) probed for tools first on the majority of their Secondary-Long and Tertiary trials.

On this evidence, the crows appear sensitive to the position of the food when deciding whether to probe for food or for tools. But do they make use of this information when choosing which tool to extract? To tackle this, we examined the length of the first non-tabletop tool used to probe for food in different conditions. We considered three candidate strategies: (1) No tool selectivity. Subjects extract and use inaccessible tools at random to probe for food. Under this strategy, in Secondary-Any and Secondary-Long conditions, the mean length of the first extracted tool used to probe for food would be expected to be 17.5 cm (the average of all the inaccessible tools: 10, 15, 20 and 25 cm), whereas in Tertiary trials the predicted average length would be 15 cm (the average of the 10, 15 and 20 cm long tools, since the 25 cm tool is out of reach). (2) A preference for long tools, irrespective of condition. Under this strategy, we would expect tools first used to probe for food to be significantly longer than the average values under random choice (see above), for all conditions. (3) Sensitivity to the demands of the task. Under this strategy, we would predict no deviation from random tool length for Secondary-Any trials, but significantly longer tools to be used in Secondary-Long and Tertiary conditions.

**Figure 5:**
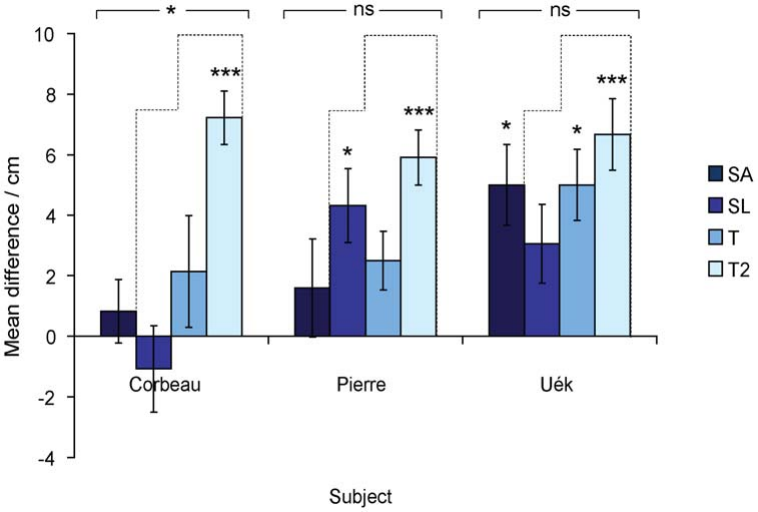
Tool selectivity in Experiment 2. Bars show the deviation (±SE) between the mean length of the tool first used by crows to probe for food (in experimental conditions) and a hypothesised mean value if subjects were picking out-of-reach tools at random. The dashed line indicates the length deviation necessary to reach the food, i.e. the deviation that would have been shown by a perfect performer. On Secondary-Any (SA) trials any of the four out-of-reach tools is correct so there is no necessary deviation. The line is lower for the Secondary-Long (SL) than for the Tertiary (T, T2) conditions because average tool length expected from random choice is longer (in SL there are four tools to choose from, leading to an expected length of 17.5 cm; in Tertiary trials, one tool is out of reach so the first choice can only be between the other three, leading to an expected length of 15 cm; for further details, see main text). *P*-values (two-tailed) from one-sample *t*-tests on the observed and hypothesized means (multiplied by the number of comparisons, *c*, for each subject; *c* = 4) are indicated by asterisks above each bar; *p*-values from Kruskal-Wallis tests are indicated above each subject (* = *p*<0.05, ** = *p*<0.01, *** = *p*<0.001). There was a significant difference in tool length between conditions for Corbeau only: post-hoc tests showed that the tools he used to probe with on Tertiary-2 trials were significantly longer than those used in all other conditions.

Figure 5 shows the deviation between the length of the extracted tools which subjects first used to probe for food, and the predicted length if they were choosing at random (see Materials and Methods). Corbeau used tools that were significantly longer than expected only in his second block of Tertiary trials, which is consistent with this subject starting with Strategy 1 (see above) and then developing selectivity by learning (in Tertiary-2). There was a significant difference in the median length of tools used by Corbeau across conditions (Kruskal-Wallis: *H* = 10.01, *df* = 3, *p*_adjusted_ = 0.02), and post-hoc Mann-Whitney *U*-tests (with p-values adjusted for multiple comparisons) confirmed that Corbeau probed with significantly longer tools in Tertiary-2 trials compared to the other three conditions (Figure 5; Secondary-Any *vs* Tertiary-2: *W* = 0.31, *p* = 0.03; Secondary-Long *vs* Tertiary-2: *W* = 35.5, *p* = 0.007; Tertiary *vs* Tertiary-2: *W* = 40.0, *p* = 0.03; all other comparisons non-significant). Pierre showed tool choice that was most consistent with Strategy 3: tool length did not differ from random expectation in Secondary-Any trials, but he used significantly longer tools in Secondary-Long and Tertiary-2 conditions (tools were also longer than the random expectation in Tertiary trials, but this was not significant at the corrected alpha level; Figure 5). There was no significant difference in tool length for Pierre across conditions, but this test approached significance and was in the predicted direction (*H* = 7.03, *df* = 3, *p*_adjusted_ = 0.07), suggesting that Pierre's behaviour was not fully insensitive to the demands of the task. Uék's behaviour supported Strategy 2: the tools that she first used to probe for food were longer than the random expectation on all experimental conditions (significantly so for all but Secondary-Long trials; Figure 5), and there was no statistical difference in the median length of tools used to probe across conditions (*H* = 2.62, *df* = 3, *p*_adjusted_ = 0.45). For two subjects, therefore, the extracted tools first used to probe for food were picked with some sensitivity to the task requirements (the third subject appeared to learn which tools to pick), but were still frequently too short to reach the food, indicating that the subjects did not anticipate the precise length of tool that was required.

**Figure 6:**
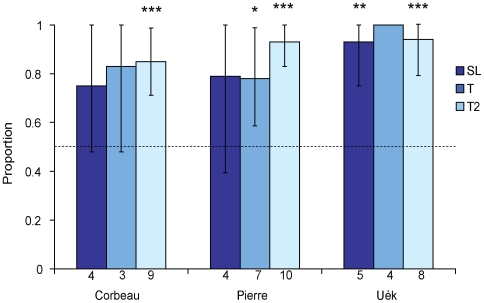
Tool ‘swaps’ in Experiment 2. Bars show the proportion of ‘positive’ tool swaps (exchanging a short tool for a longer one) compared with the expected proportion from random swapping (0.5; shown by the dashed horizontal line). Data are only for successful trials with at least one tool swap, excluding all swaps from the tabletop tool to an extracted tool (since by definition these swaps will always be positive); there were no swaps in SA trials. The number of relevant trials is displayed underneath each bar. Error bars show 95% confidence intervals and are capped at 1 (in the four relevant Tertiary trials for Uék, only positive tool swaps were made, hence the lack of an error bar). *P*-values (two-tailed) from one-sample *t*-tests of the observed and hypothesized proportions (*p*-values adjusted for each subject) are indicated by asterisks: * = *p*<0.05, ** = *p*<0.01, *** = *p*<0.001. Trial types are coded as in Figures 4 and 5, with the exception that no SA trials are presented.

To further investigate tool-selectivity, we analyzed instances of ‘tool swapping’ on those trials where subjects extracted more than one tool before their first probe for food. If they were selective about tool length, they should only make ‘positive’ swaps, i.e. swapping tools if the tool being held is too short to reach the food (therefore they should only exchange for longer tools). In contrast, if they were insensitive to tool length, we would expect a random number of ‘positive’ and ‘negative’ swaps. We only considered exchanges between extracted tools, rather than between the tabletop tool and an extracted tool, since by definition the latter swaps would always be positive. All subjects showed more positive than negative tool swaps in all conditions, significantly more than the random expectation for all subjects in Tertiary-2 trials (for data and statistics, see Figure 6). In addition, Pierre made significantly more positive swaps than expected in Tertiary trials, and Uék made significantly more positive swaps in all experimental conditions. Our analysis was limited to trials where the food was retrieved and extracted tools were swapped, and the comparatively small number of qualified trials prevents testing for statistical differences between conditions.

**Figure 7:**
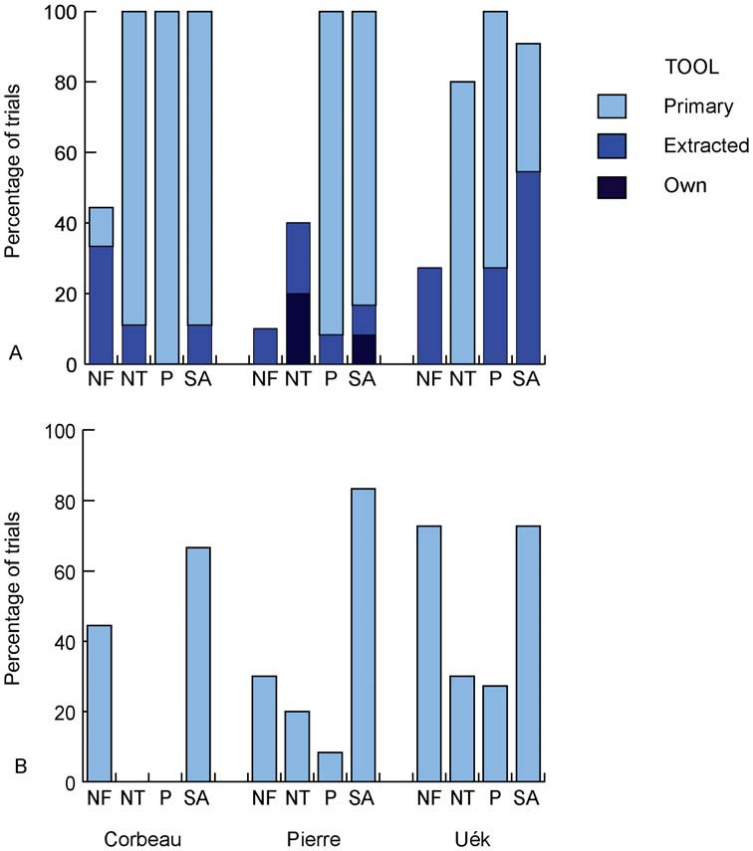
Results from control conditions in Experiment 2. Panel (A) shows the percentage of trials in which subjects probed into the food-frame, split according to which tool was used first. Panel (B) shows the percentage of trials in which a tool/object was extracted from the tool-frame. Secondary-Any (SA) trials are also displayed to allow for comparison with an experimental condition. Trial types are coded as follows: NF = No-Food; NT = No-Tools; P = Primary; SA = Secondary-Any.

Next, we examined control trials to investigate whether tools were only extracted when required. If extractions on experimental trials were goal-directed, there should have been no tool extractions when the food was within reach of the tabletop tool (Primary), or when there was no food (No-Food) or there were no usable tools in the frame (No-Tools). Similarly, if probing into the food-frame was goal-directed, there should have been no such probes with non-tool objects, or when there was no food in the frame; insertion of tools in the absence of food would question the birds' understanding of the situation, or rather, our understanding of the factors controlling the birds' behaviour. To examine this issue we compared subjects' behaviour in the three control conditions and Secondary-Any trials (as this was the simplest experimental condition) .We found a significant difference between conditions in the proportion of trials on which the food-frame was probed (Chi-square tests: Corbeau: *χ* ^2^ = 17.42, *df* = 3, *p* = 0.001; Pierre: *χ* ^2^ = 28.94, *df* = 3, *p*<0.001; Uék: *χ* ^2^ = 18.79, *df* = 3, *p*<0.001). Post-hoc examination of the standardized residuals revealed that for all subjects the No-Food condition was the most significant contributor to the chi-squared statistic. The food-frame was probed on fewer No-Food trials than predicted, although all subjects did insert an extracted tool into the (empty) food-frame on at least one trial (Figure 7A). They did not, however, insert all the tools they extracted into the food-frame, and Uék in particular, frequently took the extracted tools to other parts of the aviary, suggesting that, although crows responded to some extent appropriately to the contingencies of the task, they were motivated to extract tools *per se* (probably as play objects, or because they have value outside the experimental context). In the No-Tools condition, all subjects probed towards the food with the tabletop tool, but rarely with extracted non-tool objects (Pierre did so twice and Corbeau once). Similarly, there was a significant difference between the proportion of trials on which an object (a tool or piece of lego) was extracted (Corbeau: *χ* ^2^ = 14.94, *df* = 3, *p* = 0.002; Pierre: *χ* ^2^ = 14.78, *df* = 3, *p* = 0.002; Uék: *χ* ^2^ = 9.09, *df* = 3, *p* = 0.028). Post-hoc examination of the standardized residuals showed that, for Pierre and Corbeau, tool extractions happened more often than predicted in the Secondary-Any condition (Figure 7B).

Finally, we explored whether there was a difference between Tertiary and Tertiary-2 trials, to see whether subjects' performance on the three-tool problems improved with experience. All subjects succeeded in retrieving the food on more Tertiary-2 than Tertiary trials (Figure 4). Furthermore, the extracted tools first used by Pierre and Corbeau to probe for food were significantly longer in Tertiary-2 compared to Tertiary trials (Mann-Whitney *U*-test; Corbeau: *W* = 35.0, *p* = 0.009; Pierre: *W* = 72.5, *p* = 0.009). There was no significant difference in the median length of the tools Uék first used to probe for food between conditions (*W* = 72.0, *p* = 0.22), probably because she only probed into the food-frame with a tool shorter than 20 cm four times, and thus already had a bias for the longer tools.

#### Length-only trials

Following the completion of the Sequential trials, all three subjects were given thirty Length-Only trials where the tools were within beak range (i.e., it was unnecessary to use a tool to probe for another). All other elements of the task remained unchanged: subjects received ten trials with food at each of the previously used depths (7, 13 and 25 cm), the two short tabletop tools were present, and the same tools were available in the tool-frame. In the Sequential trials described above, subjects retrieved and used tools that were significantly longer than the average tool length predicted by random probing, but often still shorter than required to reach the food. Compared with a previous study in which two crows chose tools of appropriate length, and often longer than was necessary to reach the food [29], it therefore seems that the Sequential tool use task presented greater difficulties.

**Figure 8:**
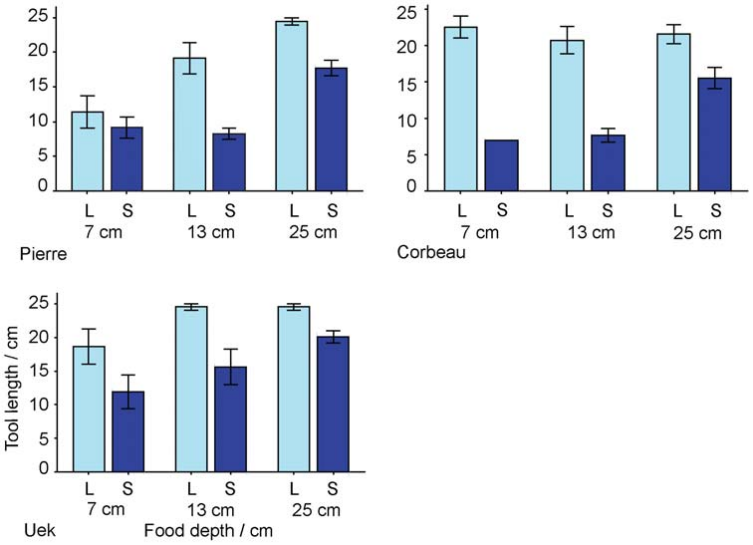
Tool selectivity in the Length-Only condition (L) compared with Sequential (S) trials (Experiment 2). Bars show mean length (±SE) of tools first used to probe for food, across the three food depths.

Our analyses revealed significant interactions between both trial type (Sequential or Length-Only) and food depth, and trial type and subject, in predicting the length of tool first used to probe for food (trial type*food depth: *F*_2,220_ = 3.92, *p* = 0.02; trial type*subject: *F*_2,220_ = 4.10, *p* = 0.02), as well as significant main effects of two of these variables (*trial type*: *F*_1,220_ = 25.03, *p* = 0.04; *food depth*: *F*_2,220_ = 29.76, *p*<0.001; *subject*: *F*_2,220_ = 2.51, *p* = 0.29). As the depth to food increased, the length of tool used to probe also increased, but tools used to probe for the food in Length-Only trials were longer than those used in Sequential trials (Figure 8). Furthermore, subjects interacted with the tabletop tools on far fewer Length-Only than Sequential trials (number of trials where a tabletop tool was the first inserted into the food-frame; Corbeau: 1/30; Pierre: 10/30; Uék: 3/10), and whilst Pierre and Uék both used a tabletop tool to probe for another tool, this happened on far fewer trials (Pierre: 2/30; Uék: 3/30). It is likely that they recognized that tools were now within beak range and therefore the attractiveness of the tabletop tools was reduced (although not entirely eliminated). Ignoring the tabletop tools, which could only reach the food on one-third of the trials, and going straight to pick out a longer tool, is in fact quite an efficient strategy.

### Concluding remarks

Our experiments provide the first demonstration, to our knowledge, that a non-human animal can spontaneously use up to three tools in a sequence to retrieve food. Most subjects showed successful, sequential tool use, using tools to extract other tools from their first trial. While previous experiments focused on *whether* subjects can perform sequential tool use, we analysed the details of their actions so as to draw inferences about the cognitive mechanisms involved.

Experiment 1 showed that crows can spontaneously perform sequential tool use. Four out of six subjects showed it reliably (another individual extracted tools but did not relate them to the task), three of them from their first trial. These three crows had prior experience with the tool apparatus before being tested, as was the case in a previous study of sequential tool use with New Caledonian crows [8]. The other three birds did not receive this pre-testing procedure, so it is interesting that only one of them acquired the skill, taking him over twenty trials to do so. However, the fact that this subject did acquire successful performance, without prior experience of picking out tools from the apparatus, demonstrates that specific pre-training on each part of the sequence was not essential. Of course, as with any test on adult animals living in natural or enriched environments, each subject is likely to have had different experiences prior to experimentation: it is possible, though unlikely, that subjects had learned the separate components of the task in which they were tested, and were thereafter chaining these learned skills [26]. There was no evidence that successful subjects were choosing which tool to extract based upon the distance to food, although the power of this negative result is low due to the small number of trials per condition. Furthermore, on the majority of their trials, two of the subjects initially probed first into the food-tube with the available, tabletop tool, which suggests either an inability to compare food depth with tool length, a lack of inhibitory control or a lack of knowledge about the requirements of the task. In a previous experiment, crows rarely probed into the food apparatus first with the available tool [8]; however, this could be simply explained by the set of extinction trials that they received before testing (the available tool was presented with food that was out of reach so subjects had experience of the inefficiency of the available tool).

 Experiment 2 enabled further insights into the underlying processes governing sequential tool use. As in Experiment 1, all three subjects showed sequential tool use on their first trials, even though the apparatus was novel and none had been given prior experience retrieving tools from the tool-frame; one subject was new to this experiment, so his first-trial tool retrieval was even more remarkable. All three subjects also successfully retrieved food in problems requiring the sequential use of three tools, and improved the efficiency of these sequences with experience. Subjects directed more of their first probes towards tools when the food was further away, demonstrating that probing for tools was not simply a result of frustration at their inability to retrieve food. Furthermore, for two of three subjects the tools that they first used to probe for food were significantly longer than would be expected if they were extracting tools randomly, indicating that they were sensitive to the required length of tool. Sensitivity to the length of tools was also evident from the subjects' tendency to exchange short tools for longer ones but not the other way round. Subjects showed flexibility in their behaviour, probing into the food-frame on fewer trials when food was absent, or could not be obtained, and extracting tools on fewer trials when they were unnecessary.

Subjects used longer tools to probe for food in Length-Only, compared to Sequential trials, i.e. when they only needed to pick out tools rather than probe for them with an available tool. Subjects also interacted with the tabletop tools on fewer Length-Only trials, indicating that they perceived this condition as distinct. During Sequential trials the tabletop tools had played an integral role in success (by extracting food or tools) on all conditions, whereas in Length-Only trials they were only suitable for retrieving the food on a third of the trials. The Length-Only trials were carried out after the Sequential experiments were complete, so their tendency to choose longer tools to probe for food might have been due to their greater experience, but it might also suggest that sequential tool use imposed extra cognitive demands, disrupting their ability to select tools based upon length [29]. Subjects not only had to recognize and respond to the depth of food, but they also had to choose whether to probe for another tool and, if so, which one. This may have had the effect of dividing their attention, a process that can affect cognitive performance [31].

In our opinion, claims for analogical reasoning based upon sequential tool use remain unjustified [8], and using sequential tool use as a benchmark of this ability is inappropriate. Reasoning (let alone analogical reasoning) is not the only cognitive mechanism to account for sequential tool use: simpler processes such as chaining may be sufficient. Conversely, animals that do possess elaborate, human-like reasoning capacities may not be identified in these experiments: errors might be made that are not due to cognitive limitations, but instead motivational, inhibitory or perceptual factors. For these reasons, we do not implicate reasoning (or a lack of it) as an explanation for our crows' behaviour, but our analyses do provide clues about the cognitive processes involved in sequential tool use. The presence of errors in subjects' sequential tool use allows us to reject the hypothesis that they were perfectly sensitive to the precise requirements of each task. However, a parsimonious alternative explanation would be that once subjects ‘discovered’ the action of tool retrieval (perhaps by displacement behaviour), they automatically performed a learned sequence of actions such as probing for food, then for tools, then for food again. Our data were not consistent with the hypothesis that subjects ‘discovered’ the out-of-reach tools in this way, nor that they then relied on a sequence of random probes for tools, followed by probes for food. We hypothesize that crows perceive general features of the task with different degree of detail, and act appropriately following this assessment. For example, crows might perceive that food is either possible or impossible to retrieve with the available tool, and that food or tools are present or absent. Under this hypothesis, the high level of incorrect first probes towards the food in Secondary-Any trials might have occurred because, at this intermediate food depth, subjects weren't sure whether the tabletop tool could retrieve it. This suggests that in the absence of clear information regarding the task they ‘give it a go’ with any available tool, and if that fails they will then search for longer tools, a strategy similar to the ‘two-stage heuristic’ proposed by Hunt and Gray to account for the choice behaviour of wild crows [32].

Our observations also highlight the importance of taking into account individual differences when considering behaviour of this complexity. Unlike in simple choice experiments, here there were many possible sequences of behaviour, so it is unsurprising that each subject showed a different strategy. The richness in our crows' behaviour makes it difficult to formulate a single model for the cognitive operations involved in sequential tool use by this (and possibly other) species. What is certain, however, is that New Caledonian crows will spontaneously use tools to probe for tools that are otherwise unavailable, and they do not need specific training to do so. What is more, although an associative process such as chaining (*sensu* Epstein *et al.* [26]) could not be ruled out for the successful subjects in Experiment 1, or in an earlier study [8], this was not the case for Experiment 2. The two subjects who had participated in Experiment 1 therefore either generalized their knowledge of the first task to the novel apparatus in Experiment 2, or they perceived it as distinct and were spontaneously able to solve it. However, the third subject in Experiment 2 used a tool to extract another tool on his very first trial, and he had no specific experience of the process, nor had he ever learned to associate the tool apparatus with tools. His behaviour in particular leaves open the possibility that crows may solve sequential tool problems by planning their actions, rather than having to build up associations by repeated experience.

While our New Caledonian crows made errors that are incompatible with fully rational planning, their performance on this task compares favourably with that of primates (including apes) in earlier studies [7], [13]. Sequential tool use is frequently assumed to be indicative of planning, a candidate for a unique characteristic of humans [33], [34]; however, together with other forms of anticipatory behaviour in corvids, it might appear that this assumption of human uniqueness is unjustified [24], [35]. The potential for sequential tool use in New Caledonian crows is likely to relate to general problem-solving abilities, rather than to an evolutionary adaptation for acquiring out-of-reach tools (which seems an unlikely scenario in their native forested habitat, where sticks are readily available). Whether these abilities are peculiar to some taxa (such as corvids, apes, and a few other groups) or more widespread, and whether they are in any way causally associated with tool use remains a matter for speculation, though the recent finding of sequential tool use in non tool-using rooks [24] suggests that the underlying cognition may have a phylogenetic origin, rather than being specifically associated with tool use. Our findings, as well as providing clues about the cognitive processes underpinning sequential tool use in New Caledonian crows, also argue for a shift away from the traditional modes of analysis: examination of the complexities in behaviour, including the types of error made, should be as important to forming conclusions about cognition as simply reporting whether or not behaviour is shown. It is now timely to run similar experiments in apes and other primates in order to determine how primate species fare under our new experimental paradigm.

## Materials and Methods

### Experiment 1

#### Subjects and housing

The subjects were six New Caledonian crows (*Corvus moneduloides*): four wild-caught adults (Betty, Barry, Kenny, Pierre; see [36] for detailed information on capture history), and two crows bred and hand-raised in captivity in 2004 (Nalik, Uék; see [37], [38] for detailed information); two were female (Betty and her daughter Uék). All subjects had participated in earlier experiments, but none had ever been required to extract non-food objects from tubes or to use tools in a sequence. Subjects were assigned to two experimental groups (‘experienced’ and ‘inexperienced’), as described in the main text. Groups were matched with respect to age and developmental history: two wild-caught and one younger, hand-reared subject in each. Betty had by far the most experimental experience; all other crows at that point had some experience. Groups were not matched for sex.

The subjects were housed in groups in indoor-outdoor aviaries (Betty and Pierre in one group, Barry and Kenny in another, and Uék and Nalik as a non-breeding pair), but tested in isolation. In their home aviaries, the crows had unlimited access to branches, sticks and children's toys of assorted sizes and shapes. As part of the standard feeding protocol, mealworms were hidden in toys and in holes in wooden logs to encourage tool use outside experimental contexts and to generally enrich their environment. We frequently observed all subjects using tools to extract hidden food.

#### Apparatus

In this experiment, both the food reward and the four potential tools were out of reach and located in tubes (Figure 2). All tubes had one blocked end and were made of transparent acrylic (food-tube: 43 cm long×5 cm diameter; tool-tubes: 30×3 cm) mounted on wooden blocks (bottom of the tubes 4.5 cm from the table). The mouth-to-mouth distance between the food-tube and the tool-tubes was 30 cm, and the angle between the two outermost tool-tubes was 100°. One (‘tabletop’) tool was placed on the table 10 cm from the open end of the food-tube. The reward was a small piece of pig's heart (∼1 g) and one waxmoth larva. In experimental trials, a piece of dowelling (2.8 cm diameter, various lengths) was placed at the back of the tool-tubes to prevent the tools from being pushed backwards during retrieval attempts. This modification was introduced after Betty and Pierre had already received, respectively, seven and five testing trials in total, where they often pushed tools out of reach by their own probing. This change did not seem to affect where the subjects chose to probe: both subjects probed for tools on their first of these trials and on subsequent trials where this was appropriate.

Pre-testing tools (see below) were pieces of bamboo skewer (diameter 0.2 cm) of different lengths. Experimental tools were made from wooden dowelling (diameter 0.6 cm). For Betty and Pierre, the tabletop tool was 6 cm long, medium-length tools were 10 cm long, and the longest tools were 20 cm long; all tools had a 2 cm long bamboo cross-piece at one end. For all other subjects, the tabletop tool was 5 cm long, medium-length tools were 12.5 cm, and the longest tools were 25 cm; the medium and long tools had four (evenly-spaced) cross-pieces, and the tabletop tool had two (one at each end). The tool lengths were changed to increase the difference between short and long tools, and the cross-pieces were added to make it easier for subjects to extract out-of-reach tools, following observations that one subject (Pierre) had difficulty with the original design.

#### General procedures

Experiments took place in a testing chamber connected to the home aviary of each subject. Subjects were isolated prior to testing, and during trials could enter and leave the experimental chamber at will. Food was removed from the home aviary at 09:00 GMT, and returned when testing ended. Prior to each testing session, the experimental room was cleared of all potential tools. Trials were performed between January 2005 and April 2006, and testing occurred between 10:00 and 19:00 GMT, with the number of trials per day and the length of the testing session depending on the subjects' willingness to participate. We terminated trials either after food retrieval, if five minutes had elapsed, or if the subject left the testing room for longer than 1 minute. The apparatus was set up out of sight of the subjects, and all trials were filmed using a mini-DV camcorder (Canon DM-MV300i, Canon DM-MV550i, or Canon XL1) from behind a tinted Perspex screen.

#### Pre-testing procedure

All birds were initially habituated to the testing room, and given trials with only the food-tube and a training tool (10–20 cm long, readily available). Once birds consistently used the tool to retrieve food (8/10 on two consecutive blocks of 10 trials), the experienced birds were presented with tool-tubes containing training tools of four lengths (see above) within beak-reach, and a food-tube with food at different depths, out of beak-reach (food could be retrieved by 1–4 of the training tools, depending on food position). After a minimum of 30 trials, there were six familiarization trials with the tools used later in testing, and food at 10–20 cm depth. The inexperienced subjects were given approximately 30 trials with food within reach of one training tool (10–20 cm), which was placed 30 cm from the mouth of the tube; first two, then four empty tool-tubes were introduced.

#### Testing procedure

To ensure the birds' motivation, food was placed just into the opening of the food-tube on the first trial of each session, so that the subject could pick it out with its beak. All birds received four trial types (see Table 1 for details of each), which differed according to the depth of the food (‘Primary’, ‘Secondary-Any’ and ‘Secondary-Long’) and the depth of the longest tool (‘Tertiary’). In addition, Barry, Kenny, Nalik, and Uék received a ‘No-Food’ condition (where the food-tube was empty), interspersed with the other trials. Betty and Pierre received three trials per condition, and the other subjects four. Conditions were pseudo-randomly ordered, with the constraint that the same condition could not occur on more than two trials in a row, and all conditions had to occur every four (for Betty and Pierre) or five trials (for the other subjects). Betty and Pierre received twelve trials in total (excluding their initial set of trials where no dowelling was present behind the tools), and the others twenty. We deliberately kept trial numbers to an absolute minimum, as we were interested in whether the crows could spontaneously solve sequential tool use tasks (as opposed to depending on training, or extensive trial-and-error learning).

After the first twenty trials, two of the inexperienced subjects (Barry and Kenny; Nalik had died) received an additional twenty trials to test whether their poor performance on the task was due to the small number of trials. Since Kenny never used one tool to retrieve another in these trials, he received a further 57 testing trials, interspersed with trials designed to promote sequential tool use (tools placed on the tabletop where tool-tubes had been located; tools placed inside the tool-tubes within reach of the beak; and tools protruding from the tool-tubes).

#### Scoring and Analysis

Scoring was done from videotapes by JHW, and all tapes were rescored by an independent observer, who was familiar with general New Caledonian crow behavior, but naïve with respect to the specific hypotheses being tested in our analyses. Concurrence was >95% for both scorers.

To address the cognitive processes underlying subjects' performance, three aspects of their behavior were examined. (i) Do subjects attend to the distance to food when choosing where to probe? (ii) Are they selective about the tools that they use to probe for food? (iii) For the one subject (Uék) who received the No-Food condition, we also looked at whether she probed the (empty) food tube and/or extracted out-of-reach tools. We investigated whether subjects attended to the position of the food by examining whether they first probed for tools or food with the tabletop tool in different conditions. Subjects who are sensitive to the distance to food should probe for it when it is within reach (Primary trials), but not when it is out of reach (experimental trials); probing for food first in experimental trials would suggest either an inability to assess the required length of the tool or to inhibit an automatic tendency to probe for food. To explore tool selectivity, we looked at the first extracted tool that the subjects used to probe for food.

The verb ‘to probe’ implies an insertion of a tool into a tube, and thus it is specifically applied to situations where a tool was in possession. Therefore, if a subject is said to probe towards the food, it is doing so with a tool. Similarly, when tools are ‘probed for’ this means with a tool, rather than simply reaching towards them.

### Experiment 2

#### Subjects and housing

We used three subjects: two from the experienced group in Experiment 1 (Pierre and Uék), and one subject (Corbeau), who had been bred and raised in captivity [37] but had never participated in problem-solving experiments, and was naïve to the task. Corbeau was housed on his own, but all housing conditions were the same as described for Experiment 1.

#### Apparatus

A new apparatus was built to address the possibility that the presentation of tools and food inside acrylic tubes in Experiment 1 had impaired the subjects' perception of their length and depth. Wooden frames were used, open at the front and top (tool-frame: l = 98 cm, w = 52 cm, d = 10 cm; food-frame: l = 30 cm, w = 21 cm, d = 11 cm; Figure 3), and these open aspects were covered in wire mesh (mesh size = 1.2 cm). The wire mesh did not completely cover the front sections of the apparatus; a 2.5 cm gap was left along the bottom to enable probing with tools and extraction of tools or food. The tool-frame was divided into four lanes (approximately 22 cm wide), and to prevent the tools from being pushed out of reach, strips of linoleum were slotted behind them (at the relevant distance). The distance between the openings of the food- and tool-frames was at least 90 cm. The pre-testing tool was a natural twig (l = 15 cm, d = 0.5 cm). Testing tools for Pierre and Corbeau were the same as those presented to most subjects in Experiment 1, in that they were made of the same dowel, and had four equally spaced skewer crosspieces. Uék was tested with natural oak twigs because she showed a strong tendency to use artificial tools for non-experimental activity (i.e., probing around her aviary) and it was very difficult to retrieve these tools from her. Unlike in Experiment 1, the out-of-reach tools were all of different lengths (10, 15, 20 and 25 cm). Testing tools were placed diagonally into each lane, at a depth of 8 cm from the open end. Two tabletop tools (5 and 7 cm long), also made of dowel and with a crosspiece at either end, were provided on every trial; only the 7 cm tool was long enough to reach food or other tools. Tabletop tools were placed midway between the tool- and food-frames, rather than closer to the food apparatus as in Experiment 1. The reward was a small piece (∼1 g) of pig's heart.

#### General procedures

Pierre was tested in a separate testing room (see general procedures for Experiment 1). Uék and Corbeau were both tested in their home aviaries, because they did not have an adjoining testing room large enough for the new apparatus. Prior to each testing session, the aviaries were cleared of all potential tools and other objects. Testing took place between August 2006 and December 2006, between 09:00 and 18:00 GMT. During trials, a small amount of the subjects' least favoured food (soaked cat biscuits) was available in the aviaries. Trial termination and recording was as described for Experiment 1.

#### Pre-testing procedure

All subjects were initially given trials with only the food-frame and a training tool (15 cm), which was readily available. Once birds consistently used the tool to retrieve food (8/10 on two consecutive blocks of 10 trials), the tool-frame was introduced (positioned as it would be during testing). Subjects received 10 trials in which a piece of meat was placed in one of the tool lanes, in a randomized order, which they could pick out with their beaks. Stones were placed into the other tool lanes. All three subjects received this familiarization, which was different to that given to both the experienced and inexperienced birds in Experiment 1. This revised protocol ensured that subjects never learned to associate the tool-frame with tools, but were still reinforced for interacting with it.

#### Testing procedure

To ensure motivation, food was placed within beak range at the opening of the food-frame, on the first trial of each session. All birds received six trial types (Table 1): Primary, Secondary-Any and Secondary-Long as in Experiment 1, plus a modified Tertiary and two control conditions (‘No-Food’ and ‘No-Tools’). In Tertiary trials, the longest tool was only reachable with the second longest out-of-reach tool, instead of all other out-of-reach tools as in Experiment 1. In No-Food trials, no reward was present, to test firstly whether tools would still be extracted (in which case the tools themselves may be reinforcing), and secondly, whether subjects would probe the empty food-frame (which would indicate that the action of probing was relatively inflexible). In No-Tools trials the tools were swapped for non-tool objects such as Lego® blocks or cork, and food was placed at an intermediate depth of 15 cm. The purpose of these trials was to see if subjects would probe for these objects, and if they picked them from the apparatus, whether they would then insert them into the food-frame.

Subjects received at least nine blocks of six trials; each condition was randomly assigned within one block. In addition, after these blocks of interspersed trials all subjects received an extra set of Tertiary trials (termed ‘Tertiary-2’), bringing the total number of these trials to at least 23. The purpose of these was to examine learning on this most challenging condition, by comparing these later trials (taking place after at least 54 exposures to the apparatus) with earlier ones.

Once sequential trials were completed, all subjects received an additional 30 ‘Length-Only’ trials. The procedure was the same as for the previous sequential tool use trials with the important difference that tools were placed within beak range at the end of each tool lane. Subjects received ten intermixed trials of each of three food depths: 7, 13 and 25 cm. These trials were carried out to determine whether there were additional cognitive demands of sequential tool use, which may have hampered tool selection.

#### Scoring and Analysis

The performance of Pierre and Corbeau was scored from videotapes by LC and re-scored by JHW for verification and analysis (concurrence was >90%; analyses use data scored by JHW). Uék's behaviour was scored from videotape by JHW. All analyses were carried out using Microsoft Excel and Minitab (version 15). All statistical tests are two-tailed, with alpha set at 0.05 (unless otherwise stated). Most analyses are carried out at a within-subject level, and therefore our inferences cannot be generalized to the species as a whole. While we acknowledge that some caution should be taken in the interpretation of such analyses, this is a general problem in animal-cognition research, where sample sizes are often small.

As in Experiment 1, we examined whether subjects retrieved the food, where they first probed, and if they exhibited tool selectivity. The target of the first probe (into the food-frame or the tool-frame) was examined using a binary logistic regression, with ‘subject’ and ‘food depth’ as factors. Food depth was used, rather than ‘condition’, because we wanted to know whether crows changed their behaviour when they perceived food to be out of reach: in this respect, there is no distinction between Secondary-Long and Tertiary trials. Tertiary-2 trials were excluded from this analysis because they were presented at the end of testing and therefore their inclusion would confound food depth with experience. Tool selectivity was examined by comparing the average length of the first extracted tool used to probe for food against a hypothesized value if the subject was picking tools at random. The expectations are described in the main text; one-sample *t*-tests (with p-values adjusted for multiple comparisons) were used for each subject to compare the observed average tool length against the hypothesized values, for each condition except No-Food and No-Tools. To evaluate whether median tool length differed between conditions we used non-parametric Kruskal-Wallis tests; where significance was reached we employed post-hoc Mann-Whitney *U*-tests (with p-values adjusted for multiple comparisons) to identify significant group differences.

We also performed three additional analyses: (i) we asked whether subjects swapped tools that they had extracted before probing for food; (ii) we examined their behaviour in control conditions, and; (iii) we investigated whether their performance on Tertiary tasks improved with experience. Tool swapping was examined by identifying all trials on which a subject extracted a tool but discarded it for another one before probing for food. The number of swaps made before the first probe was recorded, as well as the proportion of these that were ‘positive’ (i.e., where swapping resulted in increased tool length). For each subject, an average proportion was compared against the value that would be predicted if subjects were exchanging at random (i.e., 0.5), using one-sample *t*-tests (p-values adjusted for multiple comparisons). We analysed control trials with within-subject chi-squared tests to compare the number of trials on which tools/non-tool objects were extracted, as well as the number of trials on which the food-frame was probed. Where significance was reached we examined the standardized residuals to determine which cells of the contingency table significantly contributed to the chi-squared test statistic [39], comparing the size of the observed residuals to the critical values corresponding to an alpha value of 0.05. To determine whether subjects had improved their performance over the course of testing, we used Mann-Whitney *U*-tests, analyzing whether there was a difference in the median length of tools used to probe for food in Tertiary, compared to Tertiary-2 trials.

Finally, we examined the results of Length-Only trials, where tools were placed within beak range. To determine whether there was a difference in tool selection between sequential tool trials and Length-Only trials, a GLM was constructed with ‘subject’, ‘experiment’ and ‘food depth’ entered as factors.

## Supporting Information

Video S1 This video shows Betty's first exposure in Experiment 1 to the three-tool problem (Tertiary), which was her fourth testing trial in total. Correct sequence of actions: first use tabletop tool (freely available in the arena) to retrieve any of the three medium-length tools from tool-tubes (first three tool-tubes from left); then use medium-length tool to retrieve longest tool from tool-tube (fourth tool-tube from left); then use this long tool to obtain food reward from food-tube (in front). Observed behaviour: Betty does not attempt to probe for food, but immediately uses the tabletop tool to retrieve a medium-length tool. She then appears to look into the food-tube, without probing, before using the tool to extract the longest tool. Finally, she uses this tool to retrieve the reward from the food-tube. It is noteworthy that she seems to actively dispose of each tool as its role in the sequence is completed, and she also turns the tools around in order to place the cross-piece distal, where it is most effective as a hook-like instrument.
(2.96 MB MOV)

Video S2 This video shows Pierre's first exposure in Experiment 1 to a two-tool problem (Secondary-Long), which was his second testing trial in total. Correct sequence of actions: use tabletop tool (freely available in the arena) to retrieve the longest tool from the tool-tube (second tool-tube from left; the other three tubes contain medium-length tools); then use this long tool to obtain food reward from food-tube (in front). Observed behaviour: Pierre picks up the tabletop tool and quickly inserts it into the food-tube. He then uses the tabletop tool to probe for the correct, longest tool, but fails to retrieve it, and leaves the testing arena. Shortly thereafter (<1 minute; cut from video), he returns with a twig from his aviary, which he immediately uses to extract the longest tool, which he in turn uses to retrieve the reward from the food-tube.
(3.00 MB MPG)

Video S3 This video shows Uék's fifth Tertiary-2 trial in Experiment 2, which was her 67th trial in total. Correct sequence of actions: use the longer of two tabletop tools (freely available in centre of arena) to retrieve the 20 cm tool from the tool-frame (left-hand tool-lane); then use this 20 cm tool to retrieve the 25 cm tool (third tool-lane from left); then use the 25 cm tool to retrieve the food. Observed behaviour: Uék picks up the longest tabletop tool and uses it to retrieve the 20 cm tool. She uses the 20 cm tool to probe for the 25 cm tool, initially from the top of the tool-frame but then from the front. Once she has retrieved the 25 cm tool she takes it immediately to the food-frame and retrieves the food.
(5.26 MB MPG)
